# Superior Sagittal Sinus Thrombosis in a Patient With Iatrogenic Intracranial Hypotension: A Case Report

**DOI:** 10.7759/cureus.42787

**Published:** 2023-08-01

**Authors:** Sulafa Saffarini, Sally Kamil, Yazan Suradi

**Affiliations:** 1 Neurology, Wayne State University Detroit Medical Center, Detroit, USA; 2 Care of the Elderly, Glan Clwyd Hospital, Rhyl, GBR; 3 Neurology, Specialty Hospital, Amman, JOR

**Keywords:** superior sagittal sinus thrombosis, spinal surgery complication, iatrogenic intracranial hypotension, intracranial hypo-tension, cerebral venous sinus thrombosis (cvst)

## Abstract

Cerebral venous thrombosis is a rarely reported complication of iatrogenic intracranial hypotension. We discuss a rare case of a 46-year-old woman presenting with a week-long history of severe orthostatic headaches two weeks following L4-L5 microscopic discectomy for symptomatic lumbar disc herniation. Computed tomography venography of the head revealed evidence of superior sagittal sinus thrombosis while contrast-enhanced magnetic resonance imaging of the brain revealed signs of intracranial hypotension consisting of pachymeningeal enhancement, bilateral subdural hygromas, enlarged pituitary gland, effacement of the basal ambient cisterns, and low-lying cerebellar tonsils. Additional lumbar spine imaging revealed the culprit to be a large epidural fluid collection extending from the epidural space to the superficial subcutaneous fat, suggestive of a cerebrospinal fluid leak. The patient was managed with admission, bed rest, isotonic intravenous fluids, caffeine, and therapeutic dosing of low molecular weight heparin. In such cases, clinical suspicion and early recognition and management are of paramount importance to prevent devastating consequences. Management, whether conservatively or with surgical intervention, should be made on a case-by-case basis.

## Introduction

Intracranial complications following spinal procedures are rare, with an incidence of 0.4%, mostly resulting from intracranial hypotension due to durotomy and cerebrospinal fluid (CSF) leak [1]. However, the incidence of cerebral venous sinus thrombosis (CVST) due to intracranial hypotension following spinal surgeries, such as discectomy with accidental durotomy, is rarely reported in the literature [2].

The most common clinical presentation of intracranial hypotension is an orthostatic headache. Other clinical manifestations include neck and interscapular pain, nausea, vomiting, dizziness, tinnitus, diplopia, and cognitive/behavioral changes [3-4]. The most common radiological finding in intracranial hypotension is pachymeningeal gadolinium enhancement on contrast-enhanced magnetic resonance imaging (MRI). Other MRI findings include meningeal thickening, downward displacement of the cranial contents, pituitary hyperemia, and rarely subdural fluid collections (hygromas and hematomas). These findings should prompt further imaging of the spine to localize an underlying CSF leak and extradural fluid collections [5].

Management of CVST in the presence of intracranial hypotension remains unclear but the limited literature points toward targeting the intracranial hypotension initially while anticoagulation use remains controversial given the risk of developing intracranial hemorrhage in such cases. Here, we present a complex case of intracranial hypotension following L4-L5 microscopic discectomy, complicated by superior sagittal sinus thrombosis (SSST). We detail the clinical presentation, diagnosis, and management in this instance.

## Case presentation

A 46-year-old female patient was admitted to the neurosurgical service of a local private hospital for a lumbar spine microscopic discectomy. She had a longstanding history of low back pain radiating to the right lower limb with numbness along the L5 nerve root distribution. Her preoperative MRI of the lumbar spine showed a broad-based degenerative disc bulge compressing the thecal sac and the transiting cauda equina nerve roots, narrowing both lateral recesses, with minimal inferior bi-foraminal extension, resulting in moderate canal stenosis at the level of L4-L5. The patient’s other medical history was unremarkable, she was non-obese and a non-smoker. She underwent L4-L5 microscopic discectomy with an interlaminar approach. The patient’s postoperative course was uneventful, and she was discharged home two days later on oral analgesics.

Two weeks after discharge, the patient returned to the emergency department (ED) complaining of a week-long history of progressive headache. She initially had an episodic occipital headache, radiating to the neck and shoulders, primarily orthostatic, lasting for hours, and associated with neck stiffness, photophobia, phonophobia, nausea, and vomiting. There was no associated fever, rhinorrhea, lacrimation, or conjunctival injection. The headache then increased in frequency and changed character, becoming holocranial and excruciating, it eventually turned into a persistent disabling headache prior to presentation. The patient was not taking oral contraceptives or hormonal replacement agents and had no family history of thrombophilia or personal history of unexplained pregnancy loss. On presentation, she was in a normal mental state, afebrile with normal vital signs, and her neurological exam was unremarkable with no focal neurological deficits.

**Figure 1:**
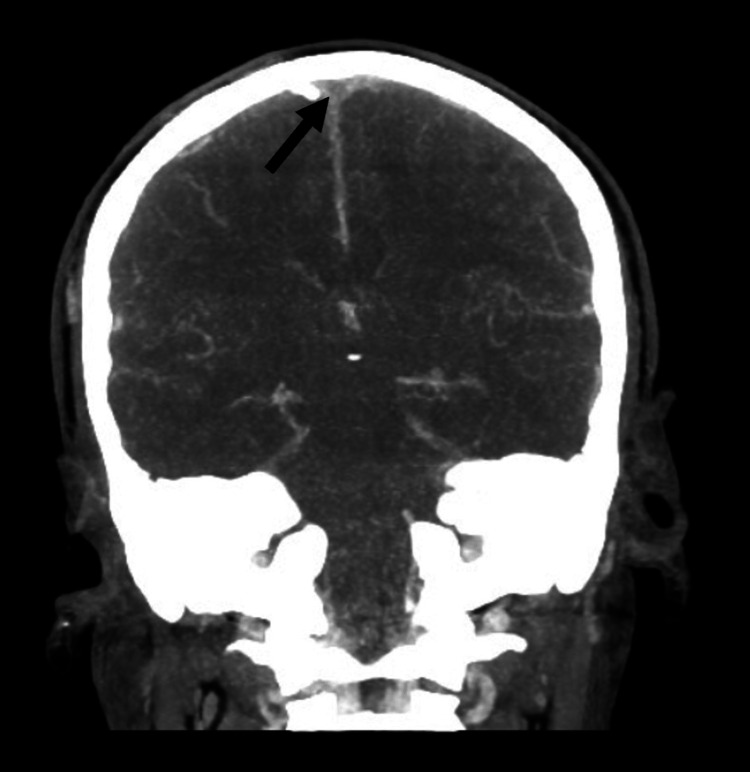
Brain CT venogram, coronal, showing non-opacified superior sagittal sinus giving the ‘empty delta sign’ (arrow)

An initial brain computed tomography (CT) scan in the ED revealed an increased density of the superior sagittal sinus with adjacent dilated cortical veins suspicious for SSST, without evidence of intracranial hemorrhage, with a normal ventricular system and no midline shift. This prompted evaluation with a brain CT-venogram, which showed a non-opacified superior sagittal sinus, giving the ‘empty delta sign’ (Figure 1), in keeping with SSST, with normal straight, sigmoid and transverse sinuses and normal deep internal cerebral veins.

**Figure 2:**
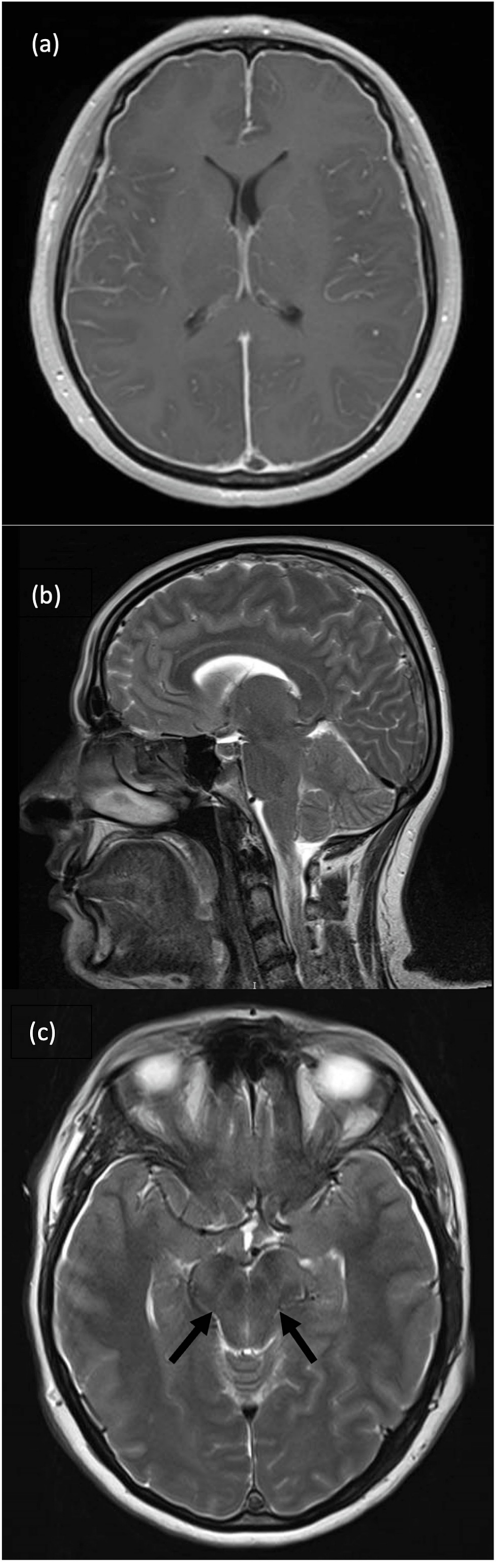
(a) Contrast-enhanced brain MRI, axial T1, showing diffuse cerebral convexity dural thickening and pachymeningeal enhancement, (b) Brain MRI, sagittal T2, showing low-lying cerebellar tonsils and enlarged pituitary gland, (c) Brain MRI, axial T2, showing effacement of the basal ambient cisterns (arrows)

The patient then underwent a contrast-enhanced brain MRI to assess for underlying venous infarcts. MRI showed evidence of intracranial hypotension, manifesting as diffuse cerebral convexity dural thickening and pachymeningeal enhancement, bilateral subdural hygromas, enlarged pituitary gland, effacement of the basal ambient cisterns, and low-lying cerebellar tonsils. Post-contrast images showed a lack of contrast opacification in the superior sagittal sinus in keeping with previously confirmed SSST (Figure 2).

**Figure 3:**
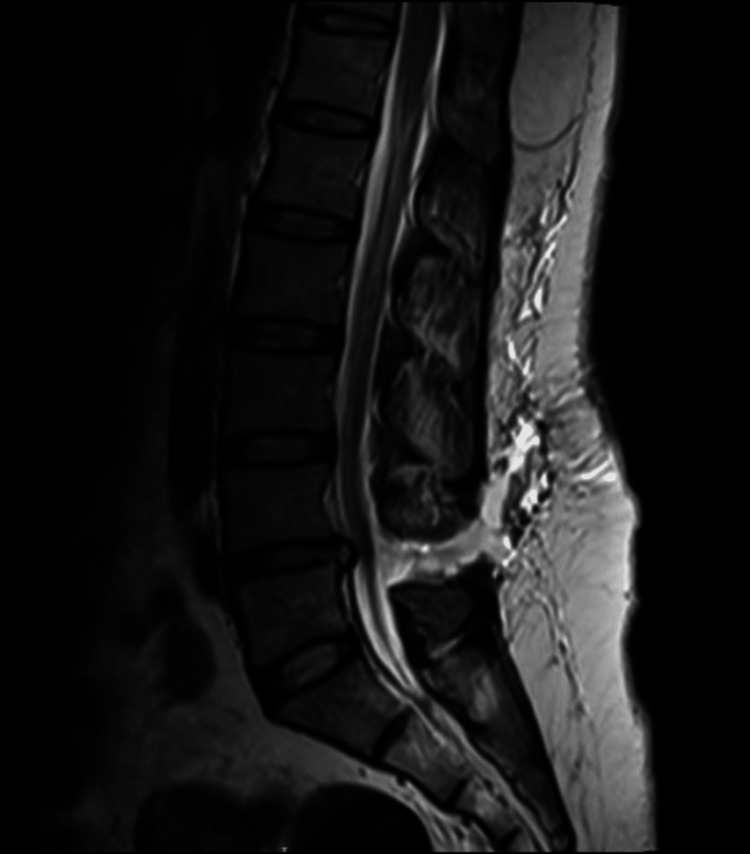
Lumbar spine MRI, sagittal T2, showing a large epidural fluid collection seen at the level of L4-L5 extending from the epidural space posteriorly through the posterior back muscles and to the superficial subcutaneous fat

Following that, a lumbar spine MRI with contrast images were obtained and revealed a large epidural fluid collection seen at the level of L4-5 extending from the epidural space posteriorly through the posterior back muscles and to the superficial subcutaneous fat, with small, localized extrathecal epidural fluid collections seen at the level of L3-L4 and L4- L5, the appearances of which were suggestive of spinal epidural CSF leak (Figure 3). 

The patient was admitted to the ward. Management consisted of regular observation with frequent neurological examination, strict horizontal bed rest, intravenous isotonic fluid hydration, paracetamol/caffeine (500 mg/65 mg) tablets, two tablets given every six hours regularly, intravenous dexamethasone for headache relief, and therapeutic dosing with low molecular weight heparin. The neurosurgical team was also consulted, and they decided to continue with conservative management and hold off on surgical intervention.

**Figure 4:**
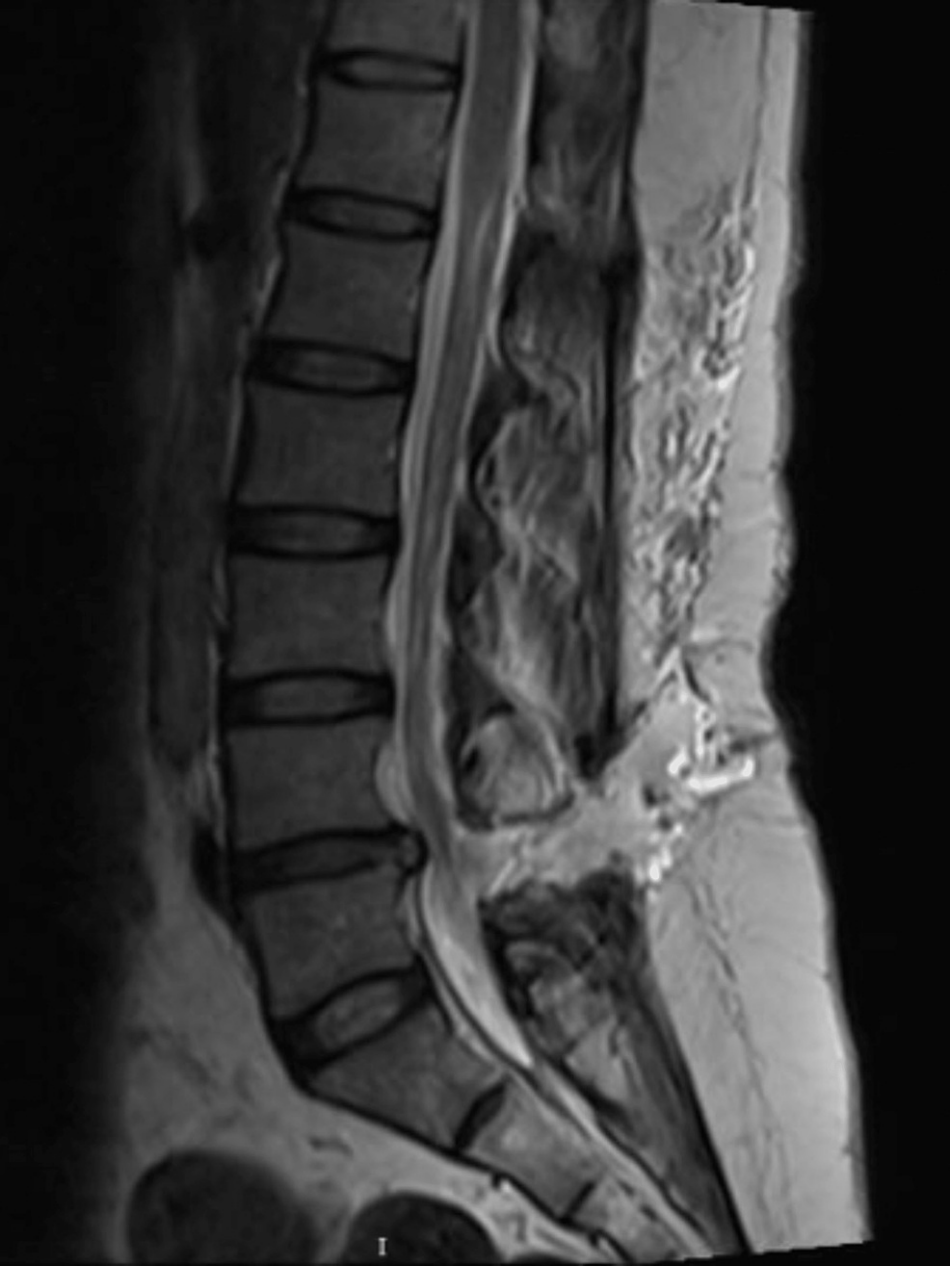
Lumbar spine MRI, sagittal T2, showing a stable epidural CSF collection

During her stay, the patient’s condition gradually improved. Her headache, phonophobia, photophobia, and neck stiffness became less pronounced over time. Follow-up lumbar spine MRI three days following admission showed no significant change regarding the previously seen large fluid collection seen at the level of L4-L5 (Figure 4). A follow-up brain MRI with IV contrast done on her fifth day of admission showed a minimal increase in the size of the bilateral subdural hygromas.

**Figure 5:**
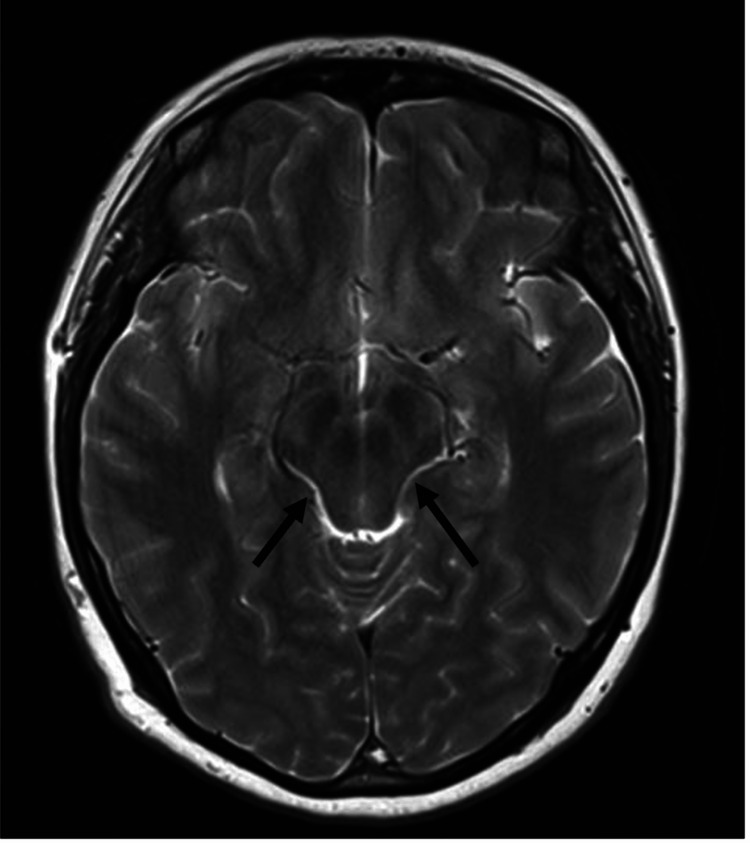
Brain MRI, axial T2, showing improvement in the effacement of the basal ambient cisterns (arrows)

She was discharged one week later on dabigatran 150 mg oral tablets twice daily, a tapered course of oral dexamethasone, ondansetron tablets, and paracetamol/caffeine tablets. Follow-up brain MRI with contrast done six days after discharge showed minimal improvement in the effacement of the basal ambient cisterns and a thrombosed right-sided cortical vein adjacent to the superior sagittal sinus while other previously reported changes remained stable (Figure 5).

## Discussion

Many reports have described the association between CVST and intracranial hypotension, whether spontaneous or induced by durotomy secondary to lumbar punctures, spinal and epidural anesthesia, and myelography [6-13]. However, only one case has been reported where CVST developed following spinal surgery [2].

A thorough understanding of CSF circulation, the cerebral venous system, the Monro-Kellie doctrine, and Virchow’s triad is of utmost importance in proposing an explanation for the pathophysiological association between intracranial hypotension and CVST. The cerebrospinal fluid is produced by the choroid plexus into the subarachnoid space and eventually drains into the venous blood of the superior sagittal sinus via arachnoid villi and arachnoid granulations [14,15]. According to the Monro-Kellie doctrine; with an intact skull, the sum of the volume of the brain plus the CSF volume plus the intracranial blood volume is constant. Therefore, a decrease in one should cause an increase in one or both of the remaining two [16]. Virchow’s triad proposes three factors contributing to the development of venous thrombosis: endothelial injury, hypercoagulability, and stasis [17].

Integrating the above knowledge can be used to explain the underlying pathophysiology that leads to the development of CVST in patients with intracranial hypotension. In patients with durotomy, CSF hypovolemia leads to an imbalance in the three intracranial volumes described by the Monroe-Kellie doctrine. Accordingly, the cerebral venous compartment compensates for this loss by venous congestion, which leads to stasis and flow turbulence. In addition, sagging of the brain occurs secondary to CSF hypovolemia, thus causing traction on the cerebral veins and sinuses; impairing the endothelial anti-thrombotic protective factors. Moreover, reduced CSF flow into the venous sinuses causes increases blood viscosity, which further decreases flow velocity. All of these factors contribute to the development of cerebral venous thrombosis.

Translating this pathophysiology into the clinical presentation, we can explain the symptomatology of our reported case. Our patient initially experienced orthostatic headaches and meningismus related to intracranial hypotension due to a CSF leak that caused meningeal traction and stimulation of meningeal nociceptive receptors. However, the change in the pattern of the headache, when it became holocranial, more frequent, and persistent is likely attributed to developing venous sinus thrombosis. Our subject had no risk factors for developing venous thrombosis i.e. smoking, use of oral contraceptives, or a personal or family history of thrombophilia, except for age and sex.

In our case, the diagnosis of intracranial hypotension was established through clinical presentation and consistent radiographic findings. A lumbar puncture was not used to document low CSF opening pressure for fear of exacerbating the underlying hypotension and potentiating the risk of further thrombosis and brain herniation.

The management of intracranial hypotension has been heavily discussed in the literature, ranging from conservative care to epidural blood patching, and open surgical intervention [3-4]. However, the management of CVST in the background of intracranial hypotension remains controversial, given the risk of developing intracranial hemorrhage in such cases.

Early identification and treatment directed at increasing the CSF pressure would theoretically prevent progression into CVST. In our case, conservative measures were taken (strict bedrest, intravenous isotonic fluid hydration, caffeine, and intravenous corticosteroids for headache relief). Regarding the SSST, our patient remained clinically stable and there was no evidence of intracranial hemorrhage on imaging. Therefore, we were more inclined to anticoagulation with a therapeutic dose of low-molecular-weight heparin in the inpatient setting, followed by oral anticoagulation (dabigatran) on discharge. Epidural blood patching and surgical intervention were not resorted to due to the fact that our patient improved on conservative measures.

## Conclusions

We report a unique case of iatrogenic intracranial hypotension complicated by SSST, both of which may have devastating consequences if not detected and managed early. In patients presenting with orthostatic headaches and meningismus following spinal surgeries, an underlying intracranial etiology should be suspected and urgent imaging should be obtained. We believe that the inciting event that leads to CVST is intracranial hypotension, and initial management should be directed toward treating it. The decision on whether to manage it conservatively or with definitive surgical repair should be tailored to each case. Overall, the reporting of CVST following spinal procedures is scarce in the literature, therefore, evidence-based algorithms for early diagnosis and treatment are yet to be recognized.
